# Change in sports activity and walking and cycling for transport since the COVID-19 pandemic – Results of the GEDA 2021 study

**DOI:** 10.25646/10666

**Published:** 2022-12-20

**Authors:** Kristin Manz, Susanne Krug

**Affiliations:** Robert Koch Institute, Berlin Department of Epidemiology and Health Monitoring

**Keywords:** SPORTS ACTIVITY, PHYSICAL ACTIVITY, CYCLING, WALKING, ADULTS, HEALTH MONITORING

## Abstract

**Background:**

Physical activity is a significant health promotion behaviour. COVID-19 pandemic mitigation measures, such as reducing social contact, closing sports facilities and working from home offices, may make it more difficult to engage in regular physical activity.

**Methods:**

The data collected between July and October 2021 from the nationally representative study German Health Update (GEDA 2021) were used. The activity behaviour is described by the change in the amount of sports activity as well as the amount of physical active transport (walking/cycling) since the beginning of the COVID-19 pandemic. The sample comprises 2,985 participants aged 18 and older.

**Results:**

A quarter of the population reduced their sports activity compared to before the COVID-19 pandemic, while 12% increased their sports activity and 38% reported no change. In terms of physical active transport, it shows that 15% reduced the amount, 17% increased it and 55% maintained it. Compared to younger adults, older adults were more likely to maintain their activity behaviour rather than reduce or increase it.

**Conclusion:**

Even before the pandemic, physical inactivity was common among the population. The high proportion of adults who reduced their sports activity during the pandemic underlines the need for effective measures to promote physical activity.

## 1. Introduction

Physical activity and sports play an important role in the prevention and treatment of a variety of non-communicable diseases [1, 2]. For example, regular physical activity reduces the risk of developing cardiovascular disease, type 2 diabetes mellitus, obesity, breast and colon cancer and depression [3, 4]. Furthermore, it is known that regular physical activity and sports activity and the associated physical fitness have a positive effect on the function of the immune system [5]. Over the course of the COVID-19 pandemic, studies have shown that physically active individuals had a lower risk of severe COVID-19 infections than less active individuals [6, 7].

The occurrence of SARS-CoV-2 infections in 2020 and 2021 had different effects on activity behaviour and can be divided into 6 phases: Phase 1 with the first COVID-19 wave and the entry into force of comprehensive containment measures (March to May 2020). This was followed by phase 2 (summer plateau – May to September 2020) with comparatively low infection levels and relaxed measures. Phase 3 and Phase 4 (October 2020 to June 2021) included the second and third COVID-19 wave and the reintroduction of containment measures, such as the obligation for employers to offer home office to employees at the end of January 2021 unless there are operational reasons not to do so (‘home office obligation’). In phase 5 (summer plateau – June to August 2021), the infection prevalence was again low, the measures were relaxed and there was a comprehensive vaccination offer. The following phase 6 was characterised by the fourth wave, which increased significantly in October 2021, and the introduction of containment measures, such as access restrictions depending on vaccination and convalescence status (the so-called 3G and 2G rules; from the end of August 2021) [8–10].

Many of the COVID-19 pandemic containment measures, especially in phases 1, 3 and 4, had the potential to reduce physical activity among the population. For example, sports facilities were closed and group sports were prohibited [11]. In addition, due to the increased work in the home office and the general request to stay at home, commutes and other distances that can be covered in a physically active way were eliminated. The obligation to be in domestic isolation due to infection or close contact with an infected person may also have had a negative impact on physical activity [12]. Day care centre and school closures challenged families and often significantly reduced the possibility for many mothers and fathers to be physically active in their free time [13, 14].

While part of the population had to give up leisure activities due to the containment measures, other parts of the population, such as men and women in short-time work, gained time that could be spent doing sports. At the same time, during the COVID-19 pandemic, new opportunities to be physically active have become established: For example, the proportion of the population in Germany using digital media for exercise (e.g. online fitness classes) was higher in autumn 2020 than before the COVID-19 pandemic [15]. Likewise, there was an increase in outdoor physical activities, such as cycling on urban green spaces [16]. However, results from the Germany-wide study ‘Examining Physical Activity and Sports Behaviour in the Face of COVID-19 Pandemic’ (SPOVID study) show that the overall proportion of adolescents and adults who reduced their sports activity during the first phase of the pandemic was significantly higher than the proportion of those who increased their sports activity (31% vs. 6%) [17]. A predominant decrease in sports activity is confirmed by the data of the German Ageing Survey (DEAS) for individuals over 45 years of age, which was collected in the summer of 2020 [18]. Reviews of international studies also conclude that physical activity decreased during the COVID-19 pandemic [19–22].

In conclusion, data on changes in physical activity in the adult population in Germany during the COVID-19 pandemic are insufficient. Especially if other areas of activity, such as physical active transport, are considered in addition to sports activity and the time period is extend beyond the first year of the pandemic.

The aim of this article is to describe the change in physical activity in terms of the amount of sports activity and active transport (walking or cycling) since the beginning of the COVID-19 pandemic, based on the nationwide study German Health Update (GEDA 2021). The temporal focus here is on the summer and autumn months of 2021.

**GEDA 2021**

Sixth follow-up survey of the German Health Update

**Data holder:** Robert Koch Institute

**Objectives:** Provision of reliable information on the health status, health behaviour and health care of the population living in Germany and their changes in the course of the SARS-CoV-2 pandemic.

**Study design:** Cross-sectional telephone survey

**Population:** German-speaking population aged 16 years and older living in private households that can be reached via landline or mobile phone

**Sampling:** Random sample of landline and mobile telephone numbers (dual-frame method) from the ADM sampling system (Arbeitskreis Deutscher Markt- und Sozialforschungsinstitute e.V.)

**Sample size:** 5,030 respondents

**Study period:** July 2021 to December 2021

**GEDA survey waves:**

► GEDA 2009
► GEDA 2010
► GEDA 2012
► GEDA 2014/2015-EHIS
► GEDA 2019/2020-EHIS
► GEDA 2021

Further information in German is available at www.geda-studie.de

## 2. Methods

### 2.1 Study design

The GEDA study is a cross-sectional survey of the German-speaking resident population aged 16 and older, which is conducted regularly as part of the nationwide health monitoring of the Robert Koch Institute (RKI). The aim of the study is to describe the health situation, health behaviour and its influencing factors, the use of prevention and care.

GEDA 2021 was conducted as the sixth follow-up survey from July to December 2021 as a telephone interview using a programmed, structured questionnaire (Computer Assisted Telephone Interview, CATI). The sampling is based on a mobile and fixed network sample (dual frame method), which allows for an almost complete coverage of the population [23, 24]. The population comprised the population aged 16 and older living in private households whose usual place of residence at the time of data collection was in Germany. The present analysis is limited to persons aged 18 and older and the survey period from mid-July to the end of October 2021 (n=2,985).

The participants were asked the following question (a) to survey the change in the amount of sport: ‘Have you changed the amount of sport you do since the start of the Corona pandemic, i.e. since March 2020?’. The four answer options were: ‘No, I do no sport’ (a1), ‘No, I do the same amount of sport overall’ (a2 ‘unchanged’), ‘Yes, I do less sport overall’ (a3 ‘reduced’), ‘Yes, I do more sport overall’ (a4 ‘increased’).

The following question (b) was asked to survey the change in physical active transport (hereafter referred to as ‘active transport’): ‘Since the start of the Corona pandemic, i.e. March 2020, have you changed the amount of walking or cycling you do for work, shopping or leisure?’. The four response categories were: ‘No, I do not walk or cycle these distances’ (b1), ‘No, I walk as much overall or cycle as much overall’ (b2 ‘unchanged’), ‘Yes, I walk less overall or cycle less overall’ (b3 ‘reduced’), ‘Yes, I walk more overall or cycle more overall’ (b4 ‘increased’).

### 2.2 Statistical methods

Gender identity was used in GEDA 2019/2020-EHIS to describe gender differences [25]. Participants were able to indicate which sex they felt they belonged to. Among participants 18 years and older, 11,959 indicated a female identity and 10,687 indicated a male identity. 62 participants indicated a different sex identity or did not provide any information. In the analyses by gender, individuals with a different gender identity or no indication are not shown. The analyses on sports activity and active transport are based on 2,985 participants aged 18 and older (1,549 women, 1,428 men and 8 individuals with a different gender identity or no information; Table 1). Of these, 2,978 participants (99.8%) have valid data on sports activities and 2,963 participants (99.3%) have valid data on active transport.

The indicators of change in physical activity, defined here as the amount of sport or active transport since the start of the COVID-19 pandemic, are presented both for the entire adult population and in relation to the active part of the population (exclusion of participants who indicated response category a1 ‘No sport’ or b1 ‘No active transport’). The number of cases for the indicator on the change in sports activity is 2,978 (response categories a1–4) and 2,337 participants (a2–4) respectively, and for the change in active transport 2,963 (b1–4) and 2,632 participants (b2–4) respectively.

Results are reported as prevalence in percent with 95% confidence interval (95% CI) separately by gender (women, men), age group in years (18–29, 30–44, 45–64, ≥65) and education status (International Standard Classification of Education, ISCED: low, medium, high education group [26]).

Multinomial logistic regression models were used to analyse the independent influence of gender, age and education on the reduction or increase of the amount of sport (a) or active transport (b) compared to unchanged activity behaviour. The dependent variables are presented in the following categories: ‘Unchanged’ (reference group), ‘Reduction’ and ‘Increase’ of the respective activity behaviour. Relative risks (RR) were calculated, which represent the ratio of two absolute risks and thus enable comparison between groups. For example, the risk for women to reduce their activity is put in relation to the risk for men to reduce their activity. An RR of 1 means that there is no difference between the groups, while a value <1 reduces the risk and a value >1 increases the risk. For the regression models, only those participants were considered who took part in sport or actively covered distances and had valid values in the variables gender, age and education. The sample size of the regression model for change in sports activity thus includes data from 2,323 participants and that of the model for change in active transport includes data from 2,616 participants.

All analyses were performed using a weighting factor that corrects for deviations of the sample from the population structure. First, a design weighting was carried out for the different selection probability (mobile and fixed network) and then an adjustment was made to official population figures with regard to age, sex, federal state and district type (as of: 31.12.2020) and in relation to education (Microcensus 2018). The analyses were carried out with Stata 17.0 using the survey procedures. A statistically significant difference between groups is assumed if the corresponding p-value is less than 0.05.

## 3. Results

###  

#### Change in sports activity since the beginning of the COVID-19 pandemic

**Figure 1:**
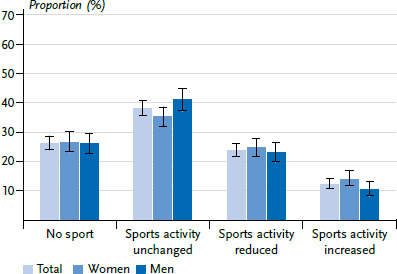
Change in sports activity since the beginning of the COVID-19 pandemic by gender (total N=2,978, women n=1,547, men n=1,423), proportion in percent with 95% confidence intervals Source: GEDA 2021

23.7% of adults reported that they had reduced their amount of sport compared to before the pandemic, while 12.1% reported that they had increased their amount of sport (Figure 1). 38.1% reported that they had not changed their amount of sport since the start of the COVID-19 pandemic. Slightly more than a quarter reported not doing any sport both before and since the start of the COVID-19 pandemic. There are no statistically significant differences between women and men with regard to the above categories of sports activity since the beginning of the COVID-19 pandemic.

If only participants who participate in sport are considered (73.9% of the adult population), the proportion of those who maintained their amount of sport since the start of the COVID-19 pandemic is 51.6% (Table 2). 32.1% of the participants who do sport reduced and 16.3% increased their amount of sport compared to the time before the pandemic. The results of the bivariate analysis show that women increased their amount of sport more often than men. The age distribution shows that adults aged 45- to 64-years and older maintained their sports activity more often than younger adults, with those under 45 years increasing their sports activity more often than older adults (Table 2). There are no significant differences between the education groups in any of the above categories (Table 2).

Regardless of gender and education, the multivariate analysis confirms that adults aged 45- to 64-years and older were more likely to maintain their sports activity compared to 18 to 29-year olds and were less likely to report a reduction or increase of their sports activity (Table 4). With regard to the increase in sports activity, the multivariate analysis also shows that women increased their sports activity more often than men, instead of maintaining it (Table 4).[Table table003]

#### Change in active transport since the beginning of the COVID-19 pandemic

**Figure 2:**
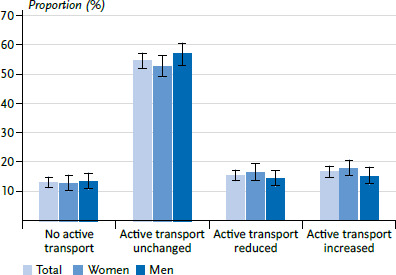
Change in active transport since the beginning of the COVID-19 pandemic by gender (total N=2,963, women n=1,536, men n=1,419), proportion in percent with 95% confidence intervals Source: GEDA 2021

In terms of the total adult population, 15.4% reported a reduction in active transport compared to the pre-pandemic period, while 16.8% reported an increase (Figure 2). The majority of all adults (54.7%) reported that they actively travel as much as they did before the pandemic. The proportion of adults who reported no active transport both before and since the beginning of the COVID-19 pandemic was 13.1%. There are no significant differences between women and men.

If only those who actively travel (86.9% of the adult population) are considered, 62.9% maintained the amount of these trips since the beginning of the COVID-19 pandemic, 17.8% reduced and 19.3% increased the amount (Table 3). The results of the bivariate analysis show that the proportion of those who reduced the volume is higher among 18- to 29-year olds than among older persons. In addition, more people in the medium compared to the low education group tended to report that they had maintained the amount of active transport. There are no statistically significant differences between women and men (Table 3).

The multivariate analysis confirms that adults aged 30 to 44 years and older were less likely than 18- to 29-year olds to reduce the active transport and more likely to maintain their pre-pandemic levels (Table 4). In addition, adults aged 65 and older were less likely to increase the amount of active transport compared to 18- to 29-year olds (Table 4). There are no statistically significant differences between women and men and between the education groups.

## 4. Discussion

The measures taken to contain the COVID-19 pandemic, such as reducing social contact, closing facilities and increasing home office working, were important steps in protecting the population from SARS-CoV-2 infection [27]. Early on in the pandemic, it was discussed that there would be profound changes in lifestyle as a result of the containment measures, which could have a negative impact on health behaviours such as physical activity [28]. However, the dynamics of the infection event and the corresponding regulations make it difficult to reach general conclusions about changes in activity behaviour in the pandemic. It is also unclear whether adults of different gender, age and education groups responded equally to containment measures in terms of their physical activity behaviour.

The present results of the GEDA 2021 study show that at the time of data collection between summer and autumn 2021, it was possible for 38% of the adult population to maintain their amount of sports activity. 12% of the population were even able to increase their sports activity. However, the proportion of people who reduced their sports activity was 24% and thus about twice as high as the proportion of those who increased their sports activity. This is particularly worrying given that a quarter of the population did not participate in sport at all and that even before the pandemic, the majority of the adult population’s physical activity level was below the recommended level [29]. More than half (55%) of the population were able to maintain the amount of active transport, 17% were able to increase it and 15% reduced it.

The results also show that adults of different ages changed the amount of sport they did differently during the pandemic. In part, there were also differences between women and men. However, there were no differences with regard to education status. With regard to the change in distances actively travelled since the beginning of the pandemic, there were also differences between adults of different ages. There were no differences between women and men or between the education groups.

A comparison of the present study results with existing literature on changes in physical activity during the COVID-19 pandemic is only possible to a limited extent, as both the survey methods (such as the questions on activity behaviour) and the times at which the studies were conducted differ. It could be shown that the physical activity of the population changed during the pandemic depending on the infection numbers and containment measures [30, 31].

In the for Germany representative SPOVID study, data on changes in physical activity behaviour of individuals aged 14 years and older were collected at the beginning of the pandemic in March and April 2020 using web-based questionnaires. The proportion of individuals who reduced their sports activity was 31% in the SPOVID study, higher than in GEDA 2021 (24%), while the proportion who increased their sports activity was lower (6% vs. 12%) [17]. One possible explanation for these deviations are the different survey times: The data collection of the SPOVID study took place in a period with comparatively strict containment measures, while at the time of the implementation of the GEDA 2021 study (summer/autumn 2021), the sports infrastructure was largely open, in compliance with infection control and hygiene measures. In addition, the proportion of vaccinated population was 63% [32] at the time of the GEDA 2021 survey, which may have encouraged parts of the population to resume previous sports habits during this phase of the pandemic.

Data on self-reported changes in sports activity during the pandemic, with a focus on the population over 45 years of age, is provided by the German Ageing Survey (DEAS), a representative survey conducted by the German Centre for Gerontology during the summer plateau 2020 [18]. According to the DEAS, more adults aged 45 and older reduced their sports activity (28%) than adults of the same age in GEDA 2021 (19%; data not shown), while the proportion of adults who did more sports activity was almost the same in both studies (8% vs. 7%; data not shown).

A higher proportion of women than men increasing their sports activity is initially surprising against the background of the existing references. A study conducted in Germany between October and November 2020 with women and men working full-time showed that women reduced their sports activity more often than men [14]. This was especially true for mothers of minor children. At the time of the present GEDA study, day care centres, schools and recreational facilities for children were widely open, which may have eased the daily burden on mothers and fathers. Mothers may have used this situation to compensate for any sport deficits from previous phases of the pandemic. This could explain the higher proportion of women with an increased amount of sport compared to before the pandemic. In addition, women and younger adults in particular used digital sports services during the pandemic, e.g. online fitness courses [15]. It is also possible that women continued the digital offers they used in the early course of the pandemic due to the higher temporal flexibility in the later course of the pandemic, when they were able to (partially) resume their original sports activities.

With regard to age differences, the results from GEDA 2021 show that adults aged 45 and older more often maintained the amount of sport than those under 45 years. On the one hand, this is positive, as older adults thus reduced their amount of sport less often, but on the other hand they also increased it significantly less often than younger adults. The SPOVID study mentioned above comes to opposite results: In March and April 2020, adults aged 30 and older were more likely to reduce their sports activity than 14- to 29-year olds [17]. It appears that older adults have found ways to return to their pre-pandemic activity levels over the course of the pandemic, possibly aided by the reopening of sports infrastructure with infection control measures in place.

Nevertheless, between summer and autumn 2021, a quarter of the adult population reported having done less sport than before the pandemic. Thus, the pandemic containment measures in place at that time may have made it difficult for at least some of the population to return to their previous levels of sports activity. Sports facilities were open to the greatest extent possible, but under so-called infection control and hygiene conditions, which included the 3G rules for indoor sports from the end of August 2021. In addition, the number of cases of individuals infected with SARS-CoV-2 increased significantly in autumn 2021, which may have led to an avoidance of contact and thus also to an avoidance of sports facilities.

According to the current state of research, there are hardly any studies so far that describe the change in active transport in Germany since the beginning of the pandemic. In a Forsa study in February 2021, the health insurance company DAK-Gesundheit asked employees who worked in a home office several times a week about a change in active transport since working predominantly in a home office [33]. The results show that more than half (54%) of the participants actively travelled less frequently. The proportion of participants who reduced active transport was significantly lower in GEDA 2021 (16%) than in the DAK study, which is presumably mainly due to the different target groups (entire adult population vs. employees in the home office) as well as the different survey times and thus different phases of the pandemic. Due to the official end of the ‘home office obligation’ in June 2021, there are fewer employees who work in a home office and can therefore actively travel to work more frequently again. This could be an explanation for the relatively low proportion of the population who reduced active transport in GEDA 2021 [9, 34]. In addition, day care centres and schools were largely open at this time and, compared to phases with more extensive restrictions, there were again more opportunities for leisure activities, which may also have led to increased active transport.

### 4.1 Strengths and limitations

Based on a nationally representative sample, this study provides results on the self-assessed change in physical activity in adulthood at the beginning of the fourth COVID-19 wave in 2021 and thus provides data for this phase of the pandemic for the first time. In addition to the change in the amount of sports activity, the change in the amount of active transport is also described. When interpreting the results, it should be noted that a bias of the results cannot be ruled out due to the reference period being relatively far in the past (time before the outbreak of the pandemic) and the associated difficulty in remembering correctly (recall bias). Furthermore, the results represent a snapshot of the time of the survey and do not include information on physical activity behaviour in earlier phases of the pandemic. Moreover, no statements can be made about the exact reasons for the change in physical activity. Besides the change in lifestyle, due to the non-pharmaceutical measures to contain the pandemic, long-term health consequences of SARS-CoV-2 infection (for example, due to Long COVID or Post COVID Syndrome) could have been another reason for the reduction in physical activity [35].

18- to 29-year olds and adults in the low education group are under-represented in the present sample, which is compensated for by conducting the analysis with a weighting factor. However, it cannot be ruled out that especially the 18- to 29-year olds or those in the low education group who participated in GEDA 2021 differed in their activity behaviour during the pandemic from those who refused to participate.

### 4.2 Conclusion

The first two years of the COVID-19 pandemic were characterised by high dynamics, both in terms of the number of infected persons and the extent of containment measures. Between July and October 2021, i.e. about one and a half years after the beginning of the pandemic, a significant proportion of the population has not returned to the amount of sports activity to which they were accustomed before the pandemic. The fact that during this period measures were relaxed to take account of 3G or 2G rules and sports facilities were open could be an indication that it is not enough to withdraw measures to contain the pandemic (for example, reopening sports infrastructure). Rather, parts of the population seem to need more support to take up sports activities again. Against the background of the generally high level of physical inactivity in the population, these results underline the need for effective measures to promote physical activity. To promote physical activity, it is recommended that so-called multi-component approaches be pursued, combining, for example, information campaigns and the design of the environment to promote physical activity [36]. In addition, cross-sectoral cooperation (for example, between the health and urban planning sectors) is required for the implementation of a large number of measures to promote physical activity.

The COVID-19 pandemic also illustrates the importance of regular and flexible monitoring of physical activity at the population level in order to detect and quantify changes in activity behaviour in a timely manner.

## Key statements

- 38% of the population did the same amount of sports in summer/fall 2021 as before the COVID-19 pandemic.
- 24% reduced their sports activity since the COVID-19 pandemic, while only 12% increased it.
- Older adults were more likely to maintain their sports activity compared to younger adults than to reduce or increase it.
- 15% of the population reduced walking and cycling for transport since the COVID-19 pandemic, 17% increased it, and 55% did not change it.
- Adults aged 18- to 29-years were more likely than older adults to report having reduced active transport.

## Figures and Tables

**Table 1:** Description of the sample by gender, age and education Source: GEDA 2021

	Numbers of cases	Proportion (unweighted)	Proportion (weighted)	Missing values
	n	%	%	n
**Total**	**2,985**	**100**	**100**	
**Gender**				8
Women	1,549	52.0	50.8	
Men	1,428	48.0	49.2	
**Age group**				0
18–29 years	261	8.7	16.2	
30–44 years	513	17.2	23.4	
45–64 years	1,145	38.4	33.7	
≥65 years	1,066	35.7	26.8	
**Education status**				12
Low	136	4.6	17.2	
education group				
Medium	1,259	42.3	57.0	
education group				
High	1,578	53.1	25.8	
education group				

**Table 2:** Change in sports activity since the beginning of the COVID-19 pandemic, by gender, age and education (total N=2,337, women n=1,214, men n=1,115) Source: GEDA 2021

	Amount of sports activity						
	Unchanged		Reduced		Increased		
	%	(95% CI)	%	(95% CI)	%	(95% CI)	p-value*
**Total**	**51.6**	**(48.4–54.7)**	**32.1**	**(29.2–35.1)**	**16.3**	**(14.1–18.9)**	
**Gender**							
Women	47.8	(43.5–52.1)	33.4	(29.4–37.6)	18.8	(15.5–22.6)	0.042
Men	55.3	(50.6–59.9)	30.9	(26.6–35.5)	13.8	(10.8–17.5)	
**Age group**							
18–29 years	39.8	(31.7–48.6)	33.3	(25.5–42.1)	26.9	(19.8–35.4)	<0.001
30–44 years	40.6	(34.1–47.5)	38.3	(31.9–45.2)	21.1	(16.1–27.1)	
45–64 years	59.9	(55.0–64.5)	28.1	(23.8–32.7)	12.1	(9.3–15.5)	
≥65 years	62.1	(56.8–67.2)	29.9	(25.2–35.0)	8.0	(5.7–11.1)	
**Education status**							
Low education group	53.0	(41.2–64.5)	26.2	(17.0–38.0)	20.8	(12.6–32.4)	0.513
Medium education group	51.3	(46.9–55.6)	33.6	(29.6–37.9)	15.1	(12.3–18.5)	
High education group	51.2	(47.2–55.1)	32.0	(28.3–35.9)	16.9	(13.8–20.5)	

CI = confidence interval

*Pearson’s chi-squared test Pearson

**Table 3 table003:** Change in active transport since the beginning of the COVID-19 pandemic, by gender, age and education (total N=2,632, women n=1,374, men n=1,251) Source: GEDA 2021

	Amount of active transport						
	Unchanged		Reduced		Increased		
	%	(95% CI)	%	(95% CI)	%	(95% CI)	p-value^*^
**Total**	**62.9**	**(60.0–65.8)**	**17.8**	**(15.5–20.2)**	**19.3**	**(17.1–21.7)**	
**Gender**							
Women	60.5	(56.4–64.4)	19.0	(15.8–22.6)	20.5	(17.5–23.9)	0.205
Men	65.7	(61.5–69.7)	16.6	(13.6–20.1)	17.7	(14.6–21.3)	
**Age group**							
18–29 years	48.1	(39.4–57.0)	29.9	(22.2–38.9)	22.0	(15.6–29.9)	<0.001
30–44 years	62.5	(56.3–68.4)	16.0	(12.0–20.9)	21.5	(16.7–27.3)	
45–64 years	67.9	(63.4–72.1)	12.9	(10.1–16.3)	19.2	(15.8–23.1)	
≥65 years	66.7	(61.9–71.2)	17.7	(14.1–22.0)	15.6	(12.3–19.5)	
**Education status**							
Low education group	53.5	(43.1–63.5)	23.4	(15.7–33.5)	23.1	(15.3–33.2)	0.055
Medium education group	66.6	(62.8–70.2)	16.2	(13.5–19.4)	17.2	(14.5–20.2)	
High education group	60.7	(56.8–64.4)	17.2	(14.4–20.4)	22.1	(19.0–25.6)	

CI = confidence interval

^*^Pearson’s chi-squared test

**Table 4:** Change in sports activity and active transport, using multinomial logistic regression, by gender, age and education Source: GEDA 2021

	Amount of sports activity (Reference group: unchanged)						Amount of active transport (Reference group: unchanged)					
	Reduced			Increased			Reduced			Increased		
Variable	RR	(95% CI)	p-value	RR	(95% CI)	p-value	RR	(95% CI)	p-value	RR	(95% CI)	p-value
**Gender**												
Women	1.29	(0.97–1.72)	0.084	1.78	(1.21–2.63)	0.004	1.31	(0.94–1.82)	0.114	1.31	(0.96–1.79)	0.088
Men	1.0			1.0			1.0			1.0		
**Age group**												
18–29 years	1.0			1.0			1.0			1.0		
30–44 years	1.08	(0.64–1.84)	0.764	0.76	(0.42–1.38)	0.370	0.4	(0.23–0.69)	0.001	0.71	(0.41–1.25)	0.240
45–64 years	0.52	(0.32–0.84)	0.008	0.28	(0.16–0.49)	<0.001	0.31	(0.19–0.52)	<0.001	0.63	(0.38–1.06)	0.081
≥65 years	0.53	(0.33–0.87)	0.011	0.18	(0.10–0.32)	<0.001	0.41	(0.24–0.68)	0.001	0.5	(0.30–0.85)	0.011
**Education status**												
Low education group	1.0			1.0			1.0			1.0		
Medium education group	1.36	(0.72–2.56)	0.343	0.78	(0.39–1.56)	0.48	0.66	(0.38–1.16)	0.153	0.61	(0.34–1.08)	0.092
High education group	1.29	(0.68–2.43)	0.435	0.88	(0.44–1.79)	0.732	0.83	(0.47–1.45)	0.51	0.91	(0.50–1.62)	0.739
n=2,323 (women n=1,210, men n=1,113)							n=2,616 (women n=1,369, men n=1,247)					

RR = Relative risk, CI = confidence interval
